# No Relation Between Cognitive Impairment, Physical Disability and Serum Biomarkers in a Cohort of Progressive Multiple Sclerosis Patients

**DOI:** 10.3390/biom15010068

**Published:** 2025-01-06

**Authors:** Bartosz Gajewski, Iwona Karlińska, Małgorzata Domowicz, Igor Bednarski, Mariola Świderek-Matysiak, Mariusz Stasiołek

**Affiliations:** Department of Neurology, Medical University of Lodz, Kopcinskiego 22, 90-153 Lodz, Poland; bartosz.gajewski@stud.umed.lodz.pl (B.G.); iwona.karlinska@umed.lodz.pl (I.K.); malgorzata.domowicz@umed.lodz.pl (M.D.); igor.bednarski@umed.lodz.pl (I.B.); mariola.swiderek-matysiak@umed.lodz.pl (M.Ś.-M.)

**Keywords:** progressive multiple sclerosis, biomarkers, cognitive impairment, progression, neurofilament light chain, chitanse-3 like-protein-1, Brief International Cognitive Assessment for Multiple Sclerosis

## Abstract

Despite significant efforts, there is still an existing need to identify diagnostic tools that would enable fast and reliable detection of the progressive stage of multiple sclerosis (MS) and help in monitoring the disease course and/or treatment effects. The aim of this prospective study in a group of people with progressive MS was to determine whether changes in the levels of selected serum biomarkers and in cognitive function may predict disease progression, and therefore refine the decision-making process in the evaluation of MS patients. Forty two (42) patients with progressive MS completed all the study procedures; the mean duration of follow-up was 12.97 months. During the observation period, serum concentration of chitinase-3 like-protein-1 (CHI3L1/YKL-40) decreased significantly in the whole study group (from 4034.95 ± 262.62 to 2866.43 ± 173.37; *p* = 0.0005), as well as in subgroups of people with secondary progressive and primary progressive MS (SPMS: from 3693.81 ± 388.68 to 2542.76 ± 256.59; *p* = 0.0207; and PPMS: from 4376.09 ± 353.27 to 3190.09 ± 233.22; *p* = 0.0089, respectively). A significant worsening of Brief International Cognitive Assessment for Multiple Sclerosis (BICAMS) scores was detected in the whole study group (from 1.18 ± 0.14 to 1.34 ± 0.15; *p* = 0.0331) as well as in the PPMS subgroup (from 1.04 ± 0.18 to 1.26 ± 0.20; *p* = 0.0216). No correlations between the analyzed molecular parameters or the results of neuropsychological tests and physical disability were observed. In conclusion, an emphasis should be placed on furthering the search for multimodal biomarkers of disease progression, especially in the PMS population, based on simultaneous analysis of several factors, such as blood biomarkers and cognitive profiles.

## 1. Introduction

Multiple sclerosis (MS) is a chronic demyelinating disease of the central nervous system (CNS), with autoimmune reaction and neurodegeneration regarded as the main pathological processes. Two main forms of clinical course are differentiated based on the presence of acute exacerbations and progression of neurological symptoms: relapsing–remitting (RRMS) and progressive (PMS) MS [1]. Furthermore, PMS is subdivided into primary progressive MS (PPMS), characterized by a gradual progression of neurological deficits from disease onset, and secondary progressive MS (SPMS), which is preceded by a relapsing–remitting course of the disease [2]. Despite many years of studies of MS, there is still an existing need for easy diagnostic tools that would enable fast diagnosis of the progressive stage of MS and help assess the efficacy of treatment. Therefore, recent studies have concentrated on finding possible molecular biomarkers of MS in cerebrospinal fluid (CSF) and blood (serum and plasma) [3].

One of the most widely studied molecular biomarkers in MS is the neurofilament light chain (NfL), which is a 68 kDa molecule belonging to the cytoskeleton proteins responsible for sustaining neuronal and axonal proportions as well as intracellular signal transmission. After the breakdown of neurons, NfLs are released into extracellular space, CSF, and ultimately into peripheral blood [4,5]. NfL release into body fluids is a physiological condition associated with the aging of neurons, and its intensity varies with sex, age and body mass index [6,7]. However, its concentration increases when excessive neuronal damage occurs; therefore, NfL is suggested as a potentially valuable tool in the assessment of patients with neurodegenerative and other CNS disorders [8]. In MS, blood and/or CSF NfL levels have been related to disease activity, physical disability, cognitive function and response to therapy. The prognostic value of NfL has been suggested in radiological isolated syndrome (RIS)/clinically isolated syndrome (CIS) conversion to definitive MS [9,10,11,12,13,14]. Only a weak diagnostic power of CSF NfL to differentiate MS from non-MS inflammatory CNS diseases was reported [15].

C-X-C Motif Chemokine Ligand 13 (CXCL-13), known as B-lymphocyte chemoattractant, is a 10.3 kDa cytokine acting as a ligand binding to CXCR5 receptors located mostly on B cells. Because CXCL-13-mediated B cells recruitment has been demonstrated as an important element of the immune pathology of MS, CXCL-13 is suggested as a biomarker of neuroinflammation [16]. This chemokine is also involved in the formation of ectopic lymphoid follicles (eLFs) in the CNS, which is a feature of CNS-compartmentalized inflammation seen most frequently in SPMS [16,17]. Associations have been described between blood and/or CSF CXCL-13 levels and disease activity, disease progression and decreased quality of life [18,19,20,21].

Another promising biomarker is chitanse-3 like-protein-1 (CHI3L1), also named YKL-40. It is a 40 kDa glycoprotein supposedly involved in inflammation and tissue remodeling [22]. YKL-40 is secreted, i.a., by astrocytes, macrophages [23] and activated microglial cells [3]. Despite its not entirely understood biological function, based on the available evidence, YKL-40 is commonly regarded as a potential biomarker of MS activity and conversion from CIS to clinically definite multiple sclerosis [24], which is in line with the modulatory role of astrocytes in the disease [25]. Peripheral blood and/or CSF levels of YKL-40 have also been reported as possible predictors of disease progression and associated with disease activity and response to therapy [26,27,28,29].

A correlation between serum/plasma and CSF levels of NfL [30,31,32,33] and YKL-40 [34] was confirmed in earlier studies, which facilitates the use of peripheral blood measurements of these parameters as biomarkers of CNS pathological processes. However, such an observation could not be made for CXCL-13. The production of this chemokine seems to be specific to either the peripheral or intrathecal compartment [35].

In view of the fact that cognitive impairment (CI) occurs even in the initial stages of MS [36], cognitive assessment has been increasingly appreciated as a crucial part of disease monitoring. Importantly, CI at early stages of MS is a prognostic marker of disability progression, and indicates an aggressive course of the disease [37]. In adults with MS, the prevalence of CI varies from 34% to 65%, and its severity and scope depend on the disease subtype [38]. In contrast to RRMS, SPMS is usually characterized by more frequent and pronounced CI, which cannot be fully and/or solely explained by longer disease duration, but rather independently, by the progressive phenotype itself [39]. Similarly, studies have shown that PPMS patients usually present with more severe CI, or with a broader spectrum of CI, than in RRMS [40]. These findings might be explained, for example, by more intense brain atrophy and cortical involvement, which belong to the typical features of PMS [39,40,41]. In general, the most affected cognition domains in MS are information processing speed (IPS), memory and executive function [40]. Some studies reported either verbal learning and verbal memory [40,42] or language and visuospatial deficits [43] as typical cognitive domains impaired in PPMS. However, other researchers did not confirm such observations [44].

Recently, an increasing number of reports on multimodal analysis of molecular biomarkers (in both blood and CSF), CI and physical disability in MS patients have been published. Such a combined analysis provides deeper insight into the complex pathophysiological processes underlying the disease and its association with clinical presentation [45]. In the present study, the authors intended to determine possible profiles of molecular and cognitive biomarkers allowing the prediction and differentiation of disease progression and thereby refine the decision-making process in the evaluation of PMS patients.

## 2. Materials and Methods

### 2.1. Patients

In this prospective longitudinal study, participants were recruited from patients of the Department of Neurology and the Neurological Outpatient Clinic at University Teaching Hospital No 1. in Lodz, Poland. This study was conducted according to the guidelines of the Declaration of Helsinki (1964) and its later amendments [46]. All study procedures were approved by the Bioethics Committee of the Medical University of Lodz (decision Nos. RNN/128/20/KE and KE/564/23). All subjects gave their informed consent before participating in this study.

Inclusion criteria encompassed a diagnosis of MS according to McDonald 2017 criteria, progressive course of the disease (PPMS or SPMS) and age ≥ 18 years [47]. SPMS diagnosis was established by the treating neurologist, and in each case reevaluated at study entry by a neurologist with >20 years of experience in diagnosing and treating MS patients, based on the definition proposed by Lorscheider et al. [48].

Exclusion criteria included the RRMS course of the disease, contraindication to MRI, diseases other than MS affecting the CNS, including infectious, inflammatory, metabolic and other neurologic disorders, and withdrawal of consent. The Beck Depression Inventory-II (BDI-II) [49] was used to screen patients for depression, and subjects with a total score over 19 points were excluded from this study. Additionally, we excluded patients with a history of drug or alcohol abuse.

### 2.2. Study Procedures

Study procedures were performed at 2 consecutive time points: at study entrance and after ≥12 months of follow-up (the median duration of the follow-up period was 12.97 months, the IQR was 12.13–14.87 months and the mean was 14.24 ± 3.96 months).

For the purpose of this study, clinical data, including demographic features, disease duration, time from diagnosis, years of education, type of therapy and EDSS score, were collected from medical histories and neurological examinations.

Blood samples were obtained from each patient, collected in 4.9 mL EDTA tubes (Sarstedt; Nümbrecht, Germany), and centrifuged at 2500× *g* for 15 min, within 30 min after sampling. Serum samples were transferred to polypropylene tubes (Eppendorf; Hamburg, Germany), stored at −80 °C, and processed in the department’s neuroimmunology laboratory in compliance with good laboratory practice. Each serum sample was tested for concentrations of NfL, CXCL-13 and YKL-40 using the direct sandwich enzyme-linked immunosorbent assay (ELISA) method. The following ELISA kits were utilized: “NF-light ELISA” (Uman Diagnostics; Umea, Sweden; detection limits for NfL: 33 pg/mL), “Human CXCL-13 ELISA” and “Human YKL-40 ELISA” (both by Biorbyt Ltd.; Cambridge, UK; detection limits for CXCL-13: 1 pg/mL and YKL-40: 10 pg/mL). Measurements were carried out in accordance with the manufacturers’ recommendations. An EPOCH (BioTek Instruments; Winooski, VT, USA) microplate spectrophotometer was used for absorbance assessment.

The cognitive function of participants was examined with a set of neuropsychological tests, including a battery for the screening assessment of cognitive functions in MS, the Polish validation of the Brief International Cognitive Assessment for Multiple Sclerosis (BICAMS, 2020) [50]. The BICAMS consists of the Symbol Digit Modalities Test (SDMT) to assess information processing speed (IPS), and the California Verbal Learning Test (CVLT) and the Brief Visuospatial Memory Test Revised (BVMT-R) to assess verbal (VM) and non-verbal memory (NVM), respectively [51]. Patients with BICAMS scores of 1, 2 or 3 points were considered cognitively impaired, while 0 points represented no significant CI [52]. Results of individual tests (SDMT, CVLT and BVMT-R) below the 5th percentile of healthy population scores were defined as CI [50]. Additionally, the Verbal Fluency Test (VFT) to assess both verbal ability (VA) and executive control ability (EA) [53,54], as well as one of the variants of the Stroop Color and Word Test (SCWT) to assess executive functions [55,56], were performed. Two versions of the VFT were applied: phonological (naming as many words starting with lower letter ‘k’ as possible in sixty seconds) and semantic (naming as many words in a given category as possible in sixty seconds; category: animals) versions. The utilized SCWT variant was also divided into two parts: SCWT-A (reading names of colors written in black print) and SCWT-B (identifying colors of printed words pertaining to the names of colors, ignoring their meaning). For the analysis, raw scores of neuropsychological tests were used, which enabled the detection of even subtle changes.

Physical disability was measured with the EDSS. For the purpose of this study, 3 different detection models of disability progression were implied: (1) an EDSS increase of 0.5; (2) an EDDS increase of 1.0 point; and (3) an EDDS increase of ≥1.0 points if the baseline EDSS was ≤5.5 points and ≥0.5 point if the baseline EDSS was >5.5 points [57].

### 2.3. Statistical Analysis

Statistical analysis was performed using Statistica software (version 13, StatSoft, Tulsa, OK, USA). Continuous variables are presented using means and standard deviations, while numerical and non-continuous variables are presented as the number of cases (N) and percentages or medians with interquartile ranges. The distribution of variables was assessed using the Shapiro–Wilk test. To compare differences between groups, the Student’s *t*-test in Welch’s modification or the Mann–Whitney U test were used. Correlations were performed using the Spearman correlation coefficient. A correlation coefficient ranging from 0.00 to 0.19 was considered as very weak, 0.20 to 0.39 as weak, 0.40 to 0.59 as moderate, 0.60 to 0.79 as strong, and 0.80 to 1.0 as very strong. A generalized linear model (GLM) with repeated measures was used to measure differences between groups in time points. To identify whether baseline parameters could predict the clinical worsening of patients, a univariate logistic regression was used. A *p*-value below 0.05 was deemed significant.

## 3. Results

### 3.1. Study Group Characteristics

**Figure 1:**
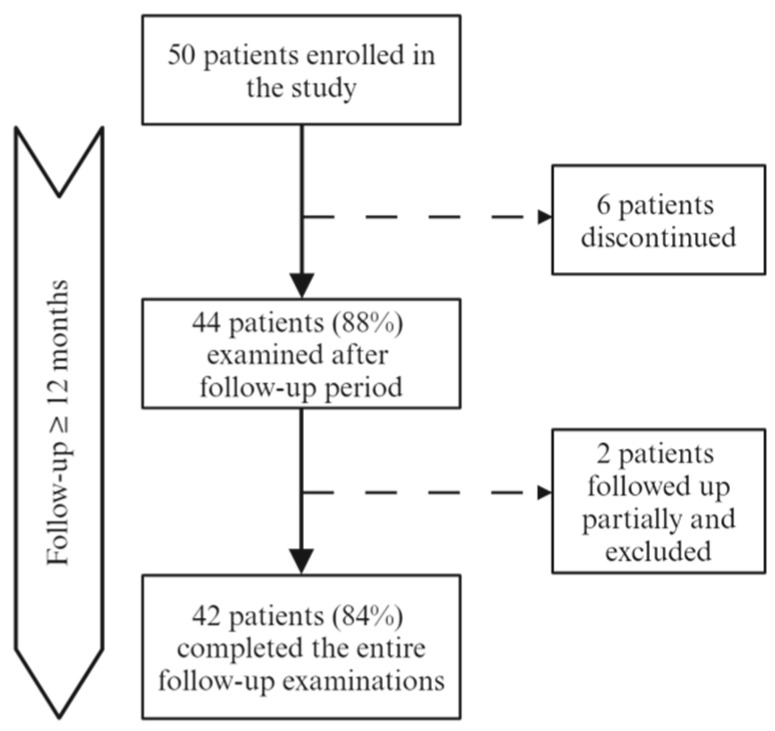
Flowchart of enrollment and follow-up of participants.

Among 50 recruited people with PMS, 6 patients withdrew from this study during the follow-up period due to personal reasons, which resulted in 44 patients (88%) being examined at the second time point (after ≥ 12 months), 2 of whom completed follow-up examinations only partially. Eventually, 42 patients who completed the entire follow-up procedure were included in the statistical analysis (see flowchart in Figure 1). Participants in this study were assigned into two groups according to PMS subtype (23 PPMS and 19 SPMS patients). Baseline characteristics of the study group are presented in Table 1. The type of disease-modifying therapy (DMT) applied at the time of recruitment and during follow-up was stratified according to the system commonly used for RRMS into moderate-efficacy (MET; teriflunomide, dimethyl fumarate, interferon-beta and glatiramer acetate) or high-efficacy DMT (HET; ocrelizumab and natalizumab) [58] (see Table 2 and Table S1 in the Supplementary Materials). However, it has to be emphasized at this point that the influence of DMTs on disease progression in MS is much lower than in the case of inflammatory activity, and only a few DMTs have been officially registered for use in progressive disease. During the observation period, no escalation of DMT was implemented and two patients discontinued DMT due to adverse events. Ten patients were enrolled in this study at the moment of starting DMT (pertains to ocrelizumab). During this study, three disease relapses were reported.

**Figure 2:**
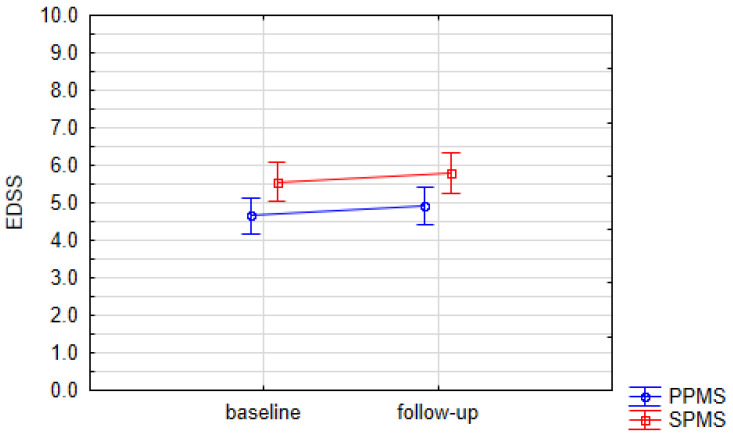
Changes in mean EDSS during study period. Data presented as means (points) with SE (whiskers). Values did not reach statistical significance. EDSS—Expanded Disability Status Scale, PPMS—primary progressive multiple sclerosis, SPMS—secondary progressive multiple sclerosis.

There was a significant difference in the disease duration and time from MS diagnosis between PPMS and SPMS groups. SPMS patients had a longer disease duration and a higher number of years from diagnosis (*p* < 0.0001) than the PPMS group. No significant differences in age and sex distribution or in years of education were found (Table 1). The majority of the studied population was receiving DMT (Table 2). A significant difference in baseline EDSS scores between PMS subgroups was demonstrated (*p* = 0.0093; Table 3). Weak positive correlations between the baseline EDSS score and both disease duration and years from diagnosis were observed (R = 0.34 and R = 0.38; *p* = 0.03 and *p* = 0.01, respectively; Table 4). However, no significant differences in EDSS between respective time points (baseline vs. follow-up) were observed in either the PPMS or the SPMS subgroup (Table 3 and Figure 2).

### 3.2. Analysis of Molecular Biomarkers

**Figure 3:**
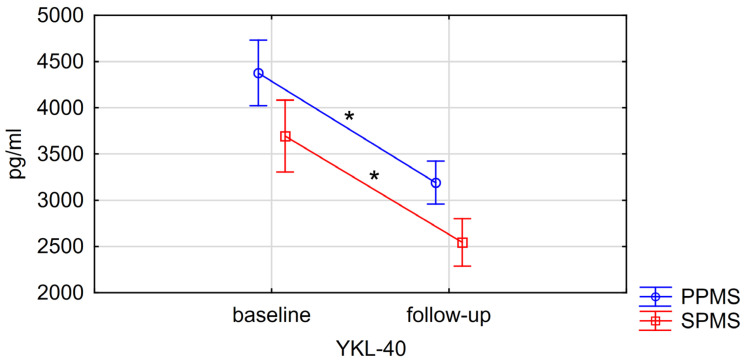
Changes in serum YKL-40 levels. Data presented as means with standard error (SE). “*” statistical significance *p* < 0.05. Figure S1A,B regarding changes in serum levels of NfL and CXCL-13, respectively, are included in Supplementary Materials. PPMS—primary progressive multiple sclerosis, SPMS—secondary progressive multiple sclerosis, NfL—neurofilament light chain, CXCL-13—C-X-C Motif Chemokine Ligand 13, YKL-40—chitanse-3 like-protein-1.

**Figure 4:**
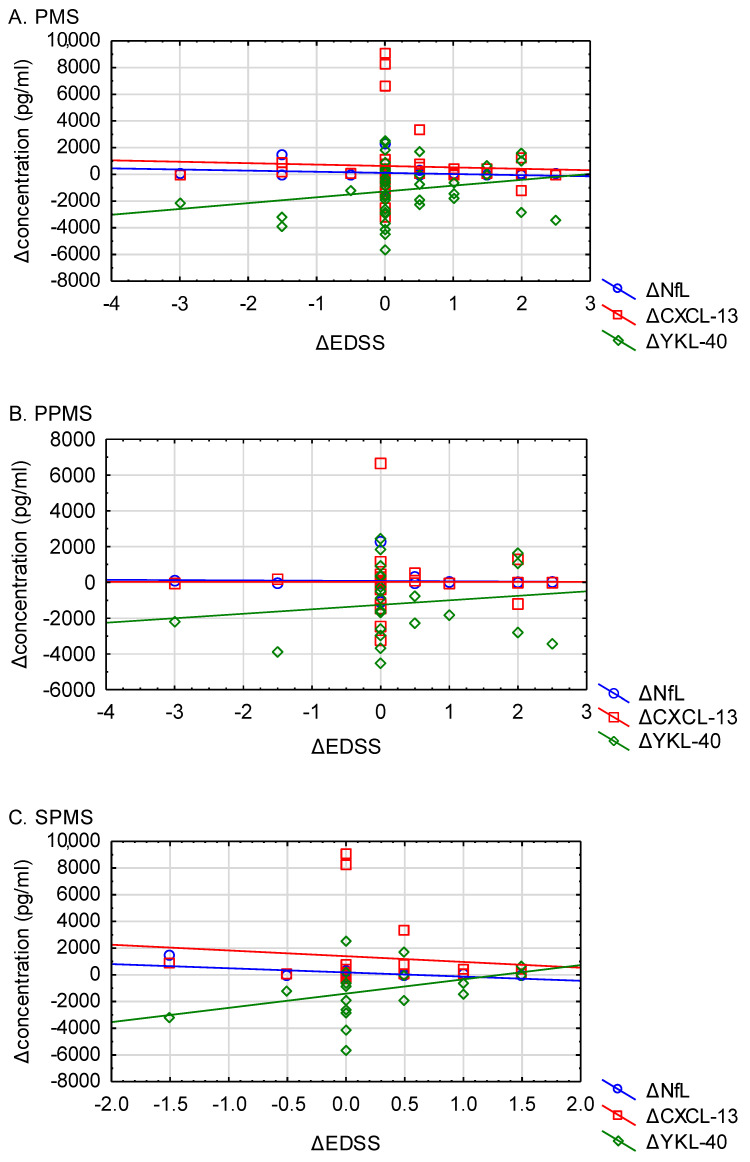
(**A**–**C**): Correlations between EDSS and serum biomarkers change. Values did not reach statistical significance. (**A**) PMS; (**B**) PPMS and (**C**) SPMS. EDSS—Expanded Disability Status Scale, PMS—progressive multiple sclerosis, PPMS—primary progressive multiple sclerosis, SPMS—secondary progressive multiple sclerosis, NfL—neurofilament light chain, CXCL-13—C-X-C Motif Chemokine Ligand 13, YKL-40—chitanse-3 like-protein-1.

The generalized linear model with repetitive measures revealed a significant decrease in YKL-40 serum concentration in the whole PMS group (from 4034.95 ± 262.62 to 2866.43 ± 173.37; *p* = 0.0005). Such a decrease was also observed in both PMS subgroups (*p* = 0.0454, Table 5 and Figure 3; Figure S1A,B). No significant differences in NfL and CXCL-13 serum concentrations were noted between study time points for the whole PMS group, nor for the PPMS and SPMS subgroups. Furthermore, no significant correlations were found between changes in serum levels of any of the biomarkers and changes in EDSS scores in the whole PMS group or in its subgroups (Table 6; Figure 4A–C).

### 3.3. Analysis of Neuropsychological Tests

**Figure 5:**
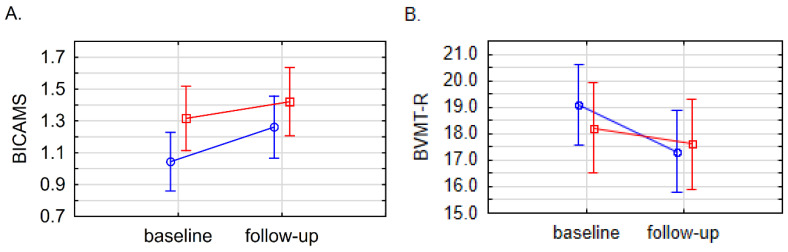

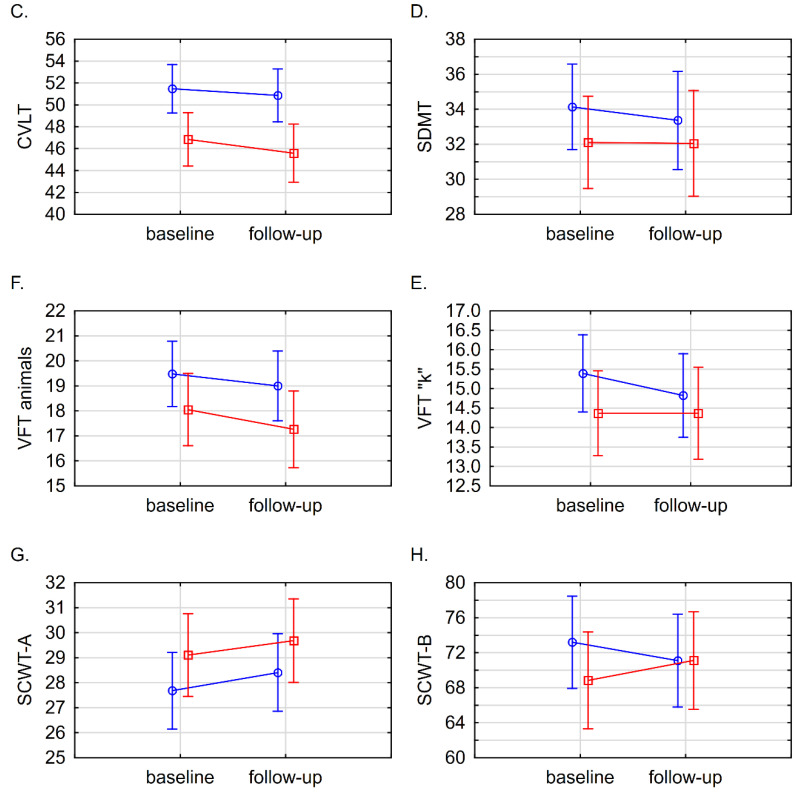
Generalized linear model with repeated measures for BICAMS (**A**), BVMT-R (**B**), CVLT (**C**), SDMT (**D**), VFT (**E**,**F**), and SCWT (**G**,**H**). Data presented as means with standard error (SE). Values did not reach statistical significance. As previously, blue indicates PPMS group and red indicates SPMS group. PPMS—primary progressive multiple sclerosis, SPMS—secondary progressive multiple sclerosis.

The generalized linear model showed a significant increase in BICAMS scores between study time points in the whole PMS (*p* = 0.0331) and PPMS groups (*p* = 0.0216), illustrating the progression of CI; however, no significant differences between PPMS and SPMS subgroups (*p* = 0.4670) were found. Regarding individual scores of SDMT, CVLT, BVMT-R, VFT and SCWT tests, no significant differences were found, either between PMS subgroups or between time points (Table 7 and Figure 5).

### 3.4. Prediction Models of Physical Disability Progression and Other Outcomes

The univariate logistic regression model did not confirm that the studied factors had potential predictive value in forecasting clinical disability progression in the PMS group and its subgroups with any of the EDSS-based definitions (Tables S2–S4). Furthermore, no correlations between analyzed molecular parameters, results of neuropsychological tests and physical disability were observed, regardless of PMS subtype and study time point.

## 4. Discussion

Various studies reported the potential usefulness of the assessment of molecular biomarkers and/or cognitive impairment in the prognosis and evaluation of disease activity and response to therapy in people with MS [45]. However, published results are not consistent, and sometimes even contradictory [59,60,61,62,63]. Notably, as compared to the number of studies of RRMS/CIS patients, data based on PMS patients seem to be much more limited. Therefore, in the current study, the prognostic potential of a set of molecular and cognitive biomarkers was evaluated in people with PMS, with additional focus on possible differences between PPMS and SPMS patients. Most importantly, our group of PMS patients did not experience significant worsening in cognitive function, which was not paralleled by the progression of neurological disability expressed as EDSS scores over a 12-month long observation. Such findings were in line with the concept of continuous “silent” progression of the disease and underscore both the insufficient sensitivity of the EDSS scale as a sole measure of progression and the need to describe and incorporate into practice better monitoring tools, e.g., tools based on a set of diverse biomarkers [64].

Many studies have indicated that changes in CXCL-13 and YKL-40 levels are not specific only to MS, but to inflammatory diseases in general [65,66,67]. Additionally, the most widely, recently studied molecular biomarker for MS—NfL—is neuron-specific and not specific to MS. Alterations in NfL levels have been studied, for example, as a biomarker in neurodegenerative diseases such as Alzheimer’s disease (AD), amyotrophic lateral sclerosis (ALS) and frontotemporal dementia, and other disorders such as stroke, CNS tumors and neuropathies [5,8,30].

In the current study, during the ≥12-month long follow-up a significant decrease in serum YKL-40 concentration in the whole PMS group was demonstrated. This might have been associated with the pathophysiology of the progressive stage of MS, characterized by a higher involvement of neurodegenerative, rather than inflammatory, processes [68]. However, in this particular PMS population, characterized by a high percentage of treated patients, the serum level of YKL-40 could also have been influenced by DMTs, as was described earlier for several individual therapies [69,70]. Canto et al. reported an increase in plasma YKL-40 levels in untreated PMS patients, whereas a trend of its decrease was observed in patients receiving interferon (IFN)-β [70]. A decrease in CSF YKL-40 levels was also reported in RRMS patients treated with fingolimod [28], natalizumab [69,71] and mitoxantrone [69]. Also, ocrelizumab was found to decrease levels of CSF YKL-40 in PPMS subjects [72]. However, data on the impact of DMTs on plasma/serum YKL-40 are scarce [73]. Interestingly, Hinsinger et al. found that YKL-40 levels in serum and CSF increased with time; nonetheless, the study concerned mostly CIS (N = 40)/RRMS (N = 66) patients and only 16 PMS subjects, the follow-up was longer (≥2 years) and no information on the applied therapy was included in the article. The authors suggested that this phenomenon could be connected with ongoing diffuse inflammation in the CNS, indicating progression of the disease [74].

Taking the above into consideration, we cannot exclude the impact of DMTs on the levels of the studied serum biomarkers.

There is an increasing body of evidence regarding a normalizing effect exerted by various DMTs on NfL [70,75,76,77,78,79] and CXCL-13 [77] levels. Our study did not reveal significant changes in serum concentrations of NfL and CXCL-13 in PMS patients during the observation period. CXCL-13, a chemokine involved, i.e., in B lymphocyte trafficking, is a biomarker of inflammatory reactions in MS. Although NfL is considered a reliable biomarker of axonal injury, its increase in body fluids has been also strongly associated with inflammatory disease activity, and to a lesser extent with neurodegenerative processes [3,16,72]. These features could at least partially explain the results obtained in our progressive MS cohort. Additionally, changes in serum levels of the included molecular biomarkers over time did not allow for differentiation of PMS subtypes (PPMS from SPMS). In a meta-analysis of 64 articles including 4071 subjects of all MS subtypes, no significant differences in CSF levels of NfL and YKL-40 between RRMS and PMS patients were reported, although the study did not differentiate between PPMS and SPMS [80]. Only 14 of 64 analyzed articles included a comparison of CSF NfL between 752 RRMS and 462 PMS subjects, and no significant difference was detected. Similar outcomes were found in the case of YKL-40 in six papers encompassing 481 RRMS and 268 PMS patients [80].

Our analysis demonstrated that serum levels of the investigated molecular biomarkers did not predict the progression of physical disability defined by any of the three different EDSS-based criteria (see the Methods section). These results remain in contrast with several previous studies showing correlation between serum NfL levels and disability progression in PMS patients [7,63]. Also, CSF levels of NfL and/or YKL-40 were suggested as predictors of neurological disability accumulation in progressive MS patients [27,62]. However, Chitnis et al., in their over 10-year long observation of patients enrolled within 5 years of disease onset in the Comprehensive Longitudinal Investigation of MS at the Brigham and Women’s Hospital (CLIMB) study, could not confirm a predictive value of serum NfL for EDSS worsening [59]. The lack of correlation of peripheral blood (serum or plasma) NfL with future disability in various groups of MS patients (including PMS subjects) was also demonstrated in other studies [60,81]. Analysis performed in a large natalizumab-treated SPMS cohort (N = 317) did not confirm the applicability of serum NfL as a biomarker of physical disability progression independent of inflammatory activity (clinical and radiological) [61]. In the light of the known association of NfL levels with the intensity of the inflammatory process in MS, it was recently suggested that the true impact of the progression of NfL levels in MS patients can be properly evaluated only after eliminating inflammatory activity with highly efficient DMT [78].

In this context, it is essential to emphasize that in numerous studies concentrated specifically on CIS and RRMS, the levels of investigated biomarkers (NfL, CXCL-13 or YKL-40) did not correlate with physical disability status or disease progression [19,82,83]. As in the case of SPMS, serum NfL in natalizumab-treated RRMS patients did not reflect progression independent of inflammatory MS activity [83].

Taking into consideration CSF analysis in relapsing forms of MS, there are multiple reports confirming the applicability of NfL, CXCL-13 and/or YKL-40 as predictive biomarkers of conversion to CDMS [84] and/or disability worsening [67,85,86,87] and/or a higher rate of disability accumulation [24]. At this point however, it is important to mention contradictory studies demonstrating the lack of value of CXCL-13 and NfL in progression prediction [19,82] and studies with better findings regarding a combined assessment of the CXCL-13 index [62] and NfL than each of those parameters alone [62].

A potential role of the investigated biomarkers in predicting conversion from RRMS to SPMS was addressed previously. In a prospective study of CIS and RRMS patients, the applicability of serum NfL in the prediction of conversion to SPMS, as well as in the early detection of patients prone to future conversion to SPMS, was reported [87]. In our study, however, SPMS patients were assessed at a later stage of the progressive phase, which can at least partially explain the differences.

There were no significant associations between the scores of neuropsychological tests and laboratory parameters or physical disability status at baseline and after the observation period in the current analysis. Furthermore, no predictive characteristics of any of the applied neuropsychological tests were established. Nonetheless, a significant worsening on the BICAMS battery, exemplifying the progression of CI in the whole PMS group, was observed. The available literature’s data in this research area for the PMS population is very limited, and the results are equivocal. In a study of PPMS patients (N = 25) cited earlier, the authors failed to show correlations between CSF NfL or YKL-40 levels and cognitive dysfunction assessed with The Rao Brief Repeatable Neuropsychological Battery [27]. Chitnis et al. did not find SDMT as predictive of worsening in the EDSS scale over a 10-year observation of MS patients [59]. Similar observations were made in CIS and RRMS cohorts. Virgilio et al. did not show CSF NfL as a predictor of CI in a cohort of newly diagnosed MS patients assessed with the BICAMS battery [12]. In a 9-year observational study of RRMS patients with higher serum and CSF NfL levels, only a weak trend of worsening, on only one of the applied tests (the California Verbal Learning Test-II; CVLT-II), was reported [88].

As opposed to our study, a recent investigation revealed a negative correlation between CSF NfL and the BICAMS z-score in a small group of PMS patients (N = 7) [10], while another recent work demonstrated a positive correlation between the CSF concentration of YKL-40 and the BICAMS in 22 PMS patients [89]. Although Pitteri et al. reported that CSF CXCL-13 levels were able to differentiate the degree of CI severity in a population of CIS, RRMS and PPMS patients, only three subjects of the latter subtype were included [90]. Finally, an association between higher serum NfL levels and the progression of CI in a SPMS cohort (N = 140) was also published [91].

Importantly, the disease duration and time since diagnosis are factors that could potentially influence cognitive deterioration [92]. Although the age of patients did not differ between our study groups, the disease duration was significantly longer in SPMS patients. Despite the lack of significant differences in cognitive performance between SPMS and PPMS patients at particular time points, in PPMS patients, we observed a significant cognitive decline that was not present in the SPMS group. This observation may be potentially attributed to differences in the dynamics of pathological processes associated with different stages of the disease. This assumption demands further research with longer follow-up.

It is also vital to account for the problem of practice effect, which is one of the most evident drawbacks of repeated neuropsychological tests [93].

Although research in this area is ongoing and results are frequently inconclusive, the molecular biomarkers of the progression in MS are one of the most important targets of modern neurology. However, the need for tools more sensitive in detecting the progression of neurodegeneration in MS has to be confronted, with multiple confounding factors associated with the complexity of disease pathology (including the changing influence of inflammatory processes) and patient-specific characteristics including age, comorbidities, applied treatments, etc. Currently, potential molecular biomarkers such as those used in this research need to be further investigated due to multiple issues, including different results obtained so far in different patient and healthy subject populations, diverse methods of measurement and difficulties defining universal normative values [3].

Conventionally applied methods of disease monitoring have another drawback, which is their inability to detect subclinical pathology, especially that associated with neurodegenerative processes in the course of MS. Molecular biomarkers and neuropsychological batteries might be of great value in this context [19], yet no perfect predictor of MS progression has been found to date.

Because most of the research in this area has been performed in RRMS and CIS patients, it is essential to include PMS patients and differentiate between its subtypes [68,94].

### Study Limitations

Taking into consideration sample sizes in other studies, and most importantly, the low prevalence of PMS (especially PPMS) in comparison to RRMS subjects, the size of our study group may be interpreted as relatively large. Nevertheless, the number of participants should be considered as a main weakness of our study, and these results should be interpreted cautiously. Also, the follow-up period of 12 months should be regarded as a basis for further observation. The relatively short follow-up might be responsible for the lack of statistically significant correlations, especially in terms of predictive values. Furthermore, the lack of neuroimaging parameters, such as brain atrophy, in the analysis should be considered as another limitation. Regarding laboratory methods employed in this study, ELISA was used due to its higher accessibility, even though a more sensitive method of proteins quantification—single molecule array (SIMOA) [95]—exists.

## 5. Conclusions

In our opinion, emphasis should be placed on further search for multimodal biomarkers based on simultaneous analysis of several factors from diverse categories, i.e., molecular biomarkers, neuropsychological tools and MRI parameters. Longer prospective longitudinal studies on larger PPMS and SPMS cohorts are warranted in order to obtain better insight into the role of molecular and neuropsychological biomarkers in the assessment and monitoring of the progressive stages of MS. We would also like to emphasize the need for non-invasive measurements of molecular factors in blood, rather than in CSF, in order to increase the applicability of these biomarkers in everyday clinical practice.

## Figures and Tables

**Table 1:** Baseline characteristics of the study group. PMS—progressive multiple sclerosis, PPMS—primary progressive multiple sclerosis, SPMS—secondary progressive multiple sclerosis. Data presented as means with SD. *p*-value refers to differences in parameters between PPMS and SPMS.

Parameter	PMS	PPMS	SPMS	*p*-Value
	(N = 42)	(N = 23)	(N = 19)	
Age [years]	53.74 ± 8.50	52.78 ± 8.38	54.89 ± 8.73	0.4402
Sex [N F, N M]	28F, 14M	15F, 8M	13F, 6M	0.8265
Years of education [years]	13.14 ± 3.22	13.48 ± 3.30	12.74 ± 3.16	0.4966
Disease duration [years]	13.64 ± 9.58	8.09 ± 6.65	20.37 ± 8.25	<0.0001
Years from diagnosis [years]	9.17 ± 8.77	4.13 ± 5.13	15.26 ± 8.45	<0.0001

**Table 2:** DMT applied in the study population. Data presented as percentages (N). DMT—disease modifying therapy, PPMS—primary progressive multiple sclerosis, SPMS—secondary progressive multiple sclerosis.

Therapy	Baseline		Follow-Up	
	PPMS	SPMS	PPMS	SPMS
moderate-efficacy	4.3% (1)	57.9% (11)	17.4% (4)	47.4% (9)
high-efficacy	52.2% (12)	21.1% (4)	56.5% (13)	26.3% (5)
no DMT	43.5% (10)	21.1% (4)	26.1% (6)	26.3% (5)

**Table 3:** EDSS scores at baseline and after follow-up period. Data presented as means with standard error (SE). EDSS—Expanded Disability Status Scale, PMS—progressive multiple sclerosis, PPMS—primary progressive multiple sclerosis, SPMS—secondary progressive multiple sclerosis. ^a^ indicates comparison between study time points calculated with *t*-test; ^b^ indicates comparison between groups and study time points calculated with generalized linear model.

EDSS	PMS (N = 42)	PPMS (N = 23)	SPMS (N = 19)	*p*-Value ^a^	*p*-Value ^b^
baseline	5.10 ± 0.18	4.65 ± 0.24	5.55 ± 0.26	0.0118	0.0093
follow-up	5.35 ± 0.18	4.91 ± 0.25	5.79 ± 0.27	0.0175	
*p*-value ^a^	0.1005	0.2911	0.1545		
*p*-value ^b^		0.9371			

**Table 4:** Correlation between baseline EDSS and demographic parameters. EDSS—Expanded Disability Status Scale, PMS—progressive multiple sclerosis, PPMS—primary progressive multiple sclerosis, SPMS—secondary progressive multiple sclerosis.

Correlation	PMS	PPMS			SPMS	
Baseline EDSS	R	*p*-value	R	*p*-value	R	*p*-value
and Age [years]	0.19	0.23	0.29	0.17	−0.10	0.69
and Years of education [years]	−0.08	0.60	−0.10	0.65	0.13	0.60
and Disease duration [years]	0.34	0.03	0.10	0.65	0.10	0.68
and Years from diagnosis [years]	0.38	0.01	0.19	0.38	0.12	0.62

**Table 5:** Serum concentrations of molecular biomarkers at baseline and after follow-up. Data presented as means with standard error (SE). PMS—progressive multiple sclerosis, PPMS—primary progressive multiple sclerosis, SPMS—secondary progressive multiple sclerosis, NfL—neurofilament light chain, CXCL-13—C-X-C Motif Chemokine Ligand 13, YKL-40—chitanse-3 like-protein-1. ^a^ indicates comparison between study time points calculated with *t*-test; ^b^ indicates comparison between groups and study time points calculated with generalized linear model.

Biomarker [pg/mL]		PMS (N = 42)	PPMS (N = 23)	SPMS (N = 19)	*p*-Value ^a^	*p*-Value ^b^
NfL	baseline	143.20 ± 71.10	194.41 ± 95.64	91.99 ± 105.23	0.4400	0.5848
	follow-up	235.79 ± 96.89	269.46 ± 130.33	202.11 ± 143.40	0.7185	
	*p*-value ^a^	0.2046	0.5104	0.1853		
	*p*-value ^b^		0.8080			
CXCL-13	baseline	920.95 ± 334.30	1485.76 ± 449.70	356.13 ± 494.77	0.0764	0.4789
	follow-up	1584.69 ± 434.66	1515.05 ± 584.69	1654.33 ± 643.30	0.8756	
	*p*-value ^a^	0.0979	0.9377	0.0513		
	*p*-value ^b^		0.0759			
YKL-40	baseline	4034.95 ± 262.62	4376.09 ± 353.27	3693.81 ± 388.68	0.2117	0.0454
	follow-up	2866.43 ± 173.37	3190.09 ± 233.22	2542.76 ± 256.59	0.0634	
	*p*-value ^a^	0.0005	0.0089	0.0207		
	*p*-value ^b^		0.9549			

**Table 6:** Correlations between EDSS and serum biomarkers change (Δ). PMS—progressive multiple sclerosis, PPMS—primary progressive multiple sclerosis, SPMS—secondary progressive multiple sclerosis, NfL—neurofilament light chain, CXCL-13—C-X-C Motif Chemokine Ligand 13, YKL-40—chitanse-3 like-protein-1.

	PMS		PPMS		SPMS	
ΔEDSS	R	*p*-value	R	*p*-value	R	*p*-value
and ΔNfL	−0.18	0.244	−0.09	0.670	−0.32	0.188
and ΔCXCL-13	0.02	0.877	0.04	0.867	0.03	0.892
and ΔYKL-40	0.23	0.146	0.09	0.668	0.41	0.082

**Table 7:** Cognitive function assessment at baseline and after follow-up. PMS—progressive multiple sclerosis, PPMS—primary progressive multiple sclerosis, SPMS—secondary progressive multiple sclerosis, BICAMS—Brief International Cognitive Assessment for Multiple Sclerosis, BVMT-R—Brief Visuospatial Memory Test Revised, CVLT—California Verbal Learning Test, SDMT—Symbol Digit Modalities Test, VFT letter ‘k’—Verbal Fluency Test phonological version, VFT animals—Verbal Fluency Test semantic version, SCWT-A—Stroop Color and Word Test part A, SCWT-B—Stroop Color and Word Test part B. ^a^ indicates comparison between study time points calculated with *t*-test; ^b^ indicates comparison between groups and study time points calculated with generalized linear model.

Test		PMS (N = 42)	PPMS (N = 23)	SPMS (N = 19)	*p*-Value ^a^	*p*-Value ^b^
BICAMS	baseline	1.18 ± 0.14	1.04 ± 0.18	1.32 ± 0.20	0.325499	0.4671
	follow-up	1.34 ± 0.15	1.26 ± 0.20	1.42 ± 0.21	0.581736	
	*p*-value ^a^	0.0331	0.0216	0.4291		
	*p*-value ^b^		0.4302			
BVMT-R sum	baseline	18.66 ± 1.14	19.09 ± 1.54	18.22 ± 1.70	0.697114	0.3861
	follow-up	17.46 ± 1.15	17.32 ± 1.55	17.61 ± 1.71	0.899304	
	*p*-value ^a^	0.0645	0.0991	0.4266		
	*p*-value ^b^		0.8965			
CVLT	baseline	49.16 ± 1.65	51.48 ± 2.21	46.84 ± 2.44	0.174226	0.7394
	follow-up	48.22 ± 1.79	50.87 ± 2.41	45.58 ± 2.66	0.150699	
	*p*-value ^a^	0.3525	0.6282	0.4229		
	*p*-value ^b^		0.1407			
SDMT	baseline	33.12 ± 1.80	34.14 ± 2.45	32.11 ± 2.63	0.564454	0.7462
	follow-up	32.71 ± 2.06	33.36 ± 2.81	32.05 ± 3.02	0.747895	
	*p*-value ^a^	0.7107	0.6175	0.9739		
	*p*-value ^b^		0.6547			
VFT letter ‘k’	baseline	14.88 ± 0.74	15.39 ± 0.99	14.37 ± 1.09	0.486471	0.5315
	follow-up	14.60 ± 0.80	14.83 ± 1.07	14.37 ± 1.18	0.773087	
	*p*-value ^a^	0.7266	0.738	0.8972		
	*p*-value ^b^		0.6171			
VFT animals	baseline	18.77 ± 0.97	19.48 ± 1.31	18.05 ± 1.44	0.467490	0.8005
	follow-up	18.13 ± 1.04	19.00 ± 1.39	17.26 ± 1.53	0.405323	
	*p*-value ^a^	0.3097	0.5721	0.3869		
	*p*-value ^b^		0.4136			
SCWT-A	baseline	28.39 ± 1.12	27.68 ± 1.53	29.11 ± 1.65	0.514741	0.9028
	follow-up	29.05 ± 1.14	28.41 ± 1.55	29.68 ± 1.67	0.565015	
	*p*-value ^a^	0.2744	0.4156	0.4841		
	*p*-value ^b^		0.5403			
SCWT-B	baseline	71.02 ± 3.82	73.19 ± 5.27	68.84 ± 5.54	0.565930	0.1096
	follow-up	71.10 ± 3.85	71.10 ± 5.30	71.11 ± 5.57	0.998947	
	*p*-value ^a^	0.9853	0.2775	0.2406		
	*p*-value ^b^		0.7751			

## Data Availability

Original contributions presented in this study are included in the article/Supplementary Materials; further inquiries can be directed to the corresponding author.

## Supplementary Materials

The following supporting information can be downloaded at: https://www.mdpi.com/article/10.3390/biom15010068/s1, Table S1: DMT distribution and treatment duration in the study population.; Figure S1: Changes in serum molecular biomarkers (A-NfL, B-CXCL-13).; Table S2: Logistic regression model for the progression of physical disability (≥1.0 EDSS) in the study group.; Table S3: Logistic regression model for the progression of physical disability (≥0.5 EDSS) in the study group.; Table S4: Logistic regression model for the progression of physical disability ≥1.0 for EDSS ≤ 5.5 or ≥0.5 for EDSS > 5.5 in the study group; Figure S2: Serum molecular biomarkers at baseline and after follow-up with regard to PMS subtype after exclusion of patients treated with ocrelizumab.
