# Supplementary material for: No Relation Between Cognitive Impairment, Physical Disability and Serum Biomarkers in a Cohort of Progressive Multiple Sclerosis Patients
Source: Biomolecules. 2025 Jan 6;15(1):68. doi: 10.3390/biom15010068 (PMC11763174; doi:10.3390/biom15010068)
Supplement: Supplementary file 1 [file biomolecules-15-00068-s001.zip › biomolecules-3330966-supplementary.pdf]

Supplementary Materials

Table S1. DMT distribution and treatment duration in the study population. Data on DMT distribution presented as percentages (N). Treatment duration for individual DMT at baseline presented as mean (SD). DMT - disease modifying therapy, PPMS - primary progressive multiple sclerosis, SPMS - secondary progressive multiple sclerosis.

Therapy	baseline		follow-up		treatment duration [years]
	PPMS	SPMS	PPMS	SPMS	
**ocrelizumab**	52.2% (12)	10.5% (2)	56.5% (13)	26.3% (5)	0.53±0.84
**natalizumab**	0.0% (0)	10.5% (2)	0.0% (0)	0.0% (0)	4.89±3.06
**teriflunomide**	0.0% (0)	15.8% (3)	4.3% (1)	10.5% (2)	1.43±0.74
**dimethyl fumarate**	0.0% (0)	10.5% (2)	13.0% (3)	5.3% (1)	4.24±0.20
**interferon-β**	0.0% (0)	21.1% (4)	0.0% (0)	21.1% (4)	4.52±4.10
**glatiramer acetate**	0.0% (0)	10.5% (2)	0.0% (0)	10.5% (2)	2.86±2.81
**cyclophosphamide**	4.3% (1)	0.0% (0)	0.0% (0)	0.0% (0)	1.36
**no DMT**	43.5% (10)	21.1% (4)	26.1% (6)	26.3% (5)	-

A.

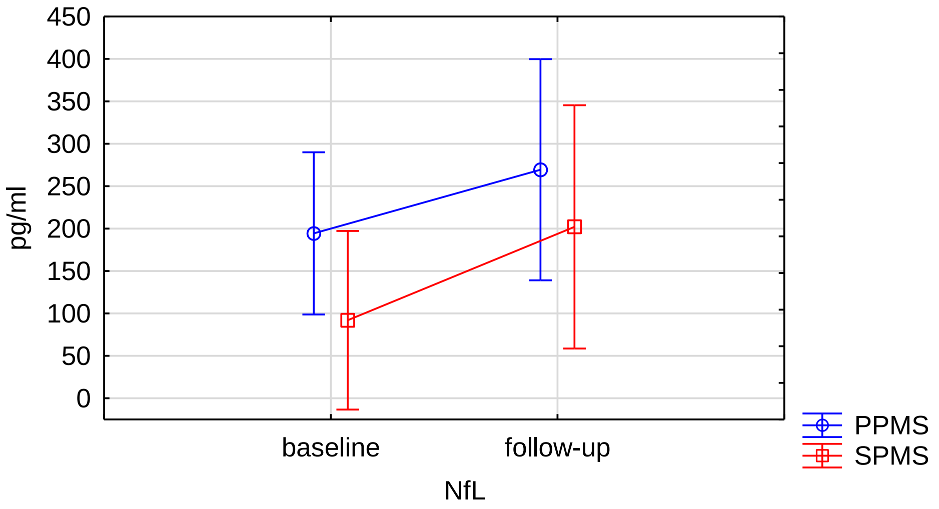

B.

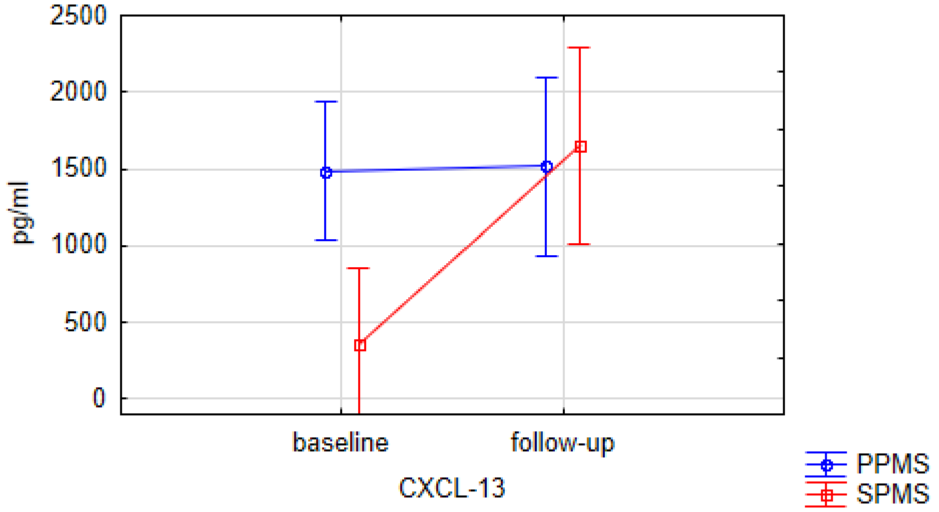

Figure S1. Changes in serum molecular biomarkers (A-NfL, B-CXCL-13). Data presented as means with standard error (SE). The values did not reach statistical significance. PPMS - primary progressive multiple sclerosis, SPMS - secondary progressive multiple sclerosis, NfL - neurofilament light chain, CXCL-13 - C-X-C Motif Chemokine Ligand 13.

Table S2. Logistic regression model for the progression of physical disability (≥1.0 EDSS) in the study group. PMS - progressive multiple sclerosis, PPMS - primary progressive multiple sclerosis, SPMS - secondary progressive multiple sclerosis, NfL - neurofilament light chain, CXCL-13 - C-X-C Motif Chemokine Ligand 13, YKL-40 - chitanse-3 like-protein-1, BICAMS - Brief International Cognitive Assessment for Multiple Sclerosis, BVMT-R - Brief Visuospatial Memory Test Revised, CVLT - California Verbal Learning Test, SDMT - Symbol Digit Modalities Test, VFT1 - Verbal Fluency Test phonological version, VFT2 - Verbal Fluency Test semantic version, SCWT-A - Stroop Color and Word Test part A, SCWT-B - Stroop Color and Word Test part B.

Group	≥1.0 EDSS	OR (95%CI)	p-value
**PMS**	NfL	0.99 (0.97; 1.01)	0.34
	CXCL-13	1.00 (1.00; 1.00)	0.32
	YKL-40	1.00 (1.00; 1.00)	0.15
	BICAMS	0.91 (0.39; 2.14)	0.83
	BVMT-R	1.00 (0.90; 1.12)	0.93
	CVLT	1.02 (0.95; 1.10)	0.56
	SDMT	0.97 (0.90; 1.04)	0.34
	VFT1	0.95 (0.80; 1.11)	0.5
	VFT2	0.98 (0.86; 1.10)	0.69
	SCWT-A	1.04 (0.94; 1.14)	0.49
	SCWT-B	1.02 (0.99; 1.05)	0.27
**SPMS**	NfL	0.99 (0.96; 1.02)	0.4
	CXCL-13	0.98 (0.96; 1.01)	0.19
	YKL-40	1.00 (1.00; 1.00)	0.13
	BICAMS	0.29 (0.05; 1.66)	0.17
	BVMT-R	1.11 (0.88; 1.41)	0.38
	CVLT	1.11 (0.97; 1.28)	0.14
	SDMT	1.15 (0.97; 1.36)	0.11
	VFT1	1.08 (0.83; 1.40)	0.56
	VFT2	1.16 (0.94; 1.43)	0.17
	SCWT-A	0.98 (0.77; 1.24)	0.86
	SCWT-B	0.97 (0.90; 1.05)	0.45
**PPMS**	NfL	0.99 (0.97; 1.02)	0.7
	CXCL-13	1.00 (1.00; 1.00)	0.45
	YKL-40	1.00 (1.00; 1.00)	0.64
	BICAMS	1.86 (0.57; 6.09)	0.31
	BVMT-R	0.97 (0.86; 1.10)	0.64
	CVLT	0.96 (0.86; 1.06)	0.4
	SDMT	0.90 (0.80; 1.00)	0.06
	VFT1	0.86 (0.68; 1.08)	0.19
	VFT2	0.83 (0.67; 1.03)	0.09
	SCWT-A	1.05 (0.94; 1.17)	0.4

	SCWT-B	1.03 (0.99; 1.07)	0.12

Table S3. Logistic regression model for the progression of physical disability (≥0.5 EDSS) in the study group. PMS - progressive multiple sclerosis, PPMS - primary progressive multiple sclerosis, SPMS - secondary progressive multiple sclerosis, NfL - neurofilament light chain, CXCL-13 - C-X-C Motif Chemokine Ligand 13, YKL-40 - chitanse-3 like-protein-1, BICAMS - Brief International Cognitive Assessment for Multiple Sclerosis, BVMT-R - Brief Visuospatial Memory Test Revised, CVLT - California Verbal Learning Test, SDMT - Symbol Digit Modalities Test, VFT1 - Verbal Fluency Test phonological version, VFT2 - Verbal Fluency Test semantic version, SCWT -A - Stroop Color and Word Test part A, SCWT-B - Stroop Color and Word Test part B.

Group	≥0.5 EDSS	OR (95%CI)	p-value
**PMS**	NfL	0.99 (0.97; 1.01)	0.15
	CXCL-13	1.00 (1.00; 1.00)	0.13
	YKL-40	1.00 (1.00; 1.00)	0.12
	BICAMS	0.61 (0.28; 1.34)	0.22
	BVMT-R	1.02 (0.93; 1.12)	0.7
	CVLT	1.05 (0.98; 1.12)	0.19
	SDMT	1.00 (0.95; 1.06)	0.88
	VFT1	1.00 (0.87; 1.14)	0.94
	VFT2	1.02 (0.91; 1.13)	0.78
	SCWT-A	1.02 (0.93; 1.12)	0.63
	SCWT-B	1.01 (0.98; 1.04)	0.39
**SPMS**	NfL	0.98 (0.94; 1.01)	0.19
	CXCL-13	1.00 (0.99; 1.00)	0.19
	YKL-40	1.00 (1.00; 1.00)	0.14
	BICAMS	0.30 (0.07; 1.29)	0.1
	BVMT-R	1.06 (0.89; 1.26)	0.53
	CVLT	1.08 (0.98; 1.20)	0.12
	SDMT	1.14 (0.99; 1.31)	0.07
	VFT1	1.14 (0.91; 1.43)	0.26
	VFT2	1.11 (0.94; 1.31)	0.24
	SCWT-A	1.03 (0.85; 1.26)	0.74
	SCWT-B	1.00 (0.95; 1.05)	0.92
**PPMS**	NfL	1.00 (0.98; 1.02)	0.65
	CXCL-13	1.00 (1.00; 1.00)	0.26
	YKL-40	1.00 (1.00; 1.00)	0.6
	BICAMS	0.92 (0.32; 2.60)	0.87
	BVMT-R	1.00 (0.90; 1.12)	0.94
	CVLT	1.02 (0.93; 1.12)	0.72
	SDMT	0.96 (0.90; 1.04)	0.32
	VFT1	0.91 (0.75; 1.10)	0.33
	VFT2	0.95 (0.82; 1.10)	0.5
	SCWT-A	1.02 (0.92; 1.13)	0.74
	SCWT-B	1.02 (0.98; 1.05)	0.28

Table S4. Logistic regression model for the progression of physical disability ≥1.0 for EDSS ≤5.5 or ≥0.5 for EDSS >5.5 in the study group. PMS - progressive multiple sclerosis, PPMS - primary progressive multiple sclerosis, SPMS - secondary progressive multiple sclerosis, NfL - neurofilament light chain, CXCL-13 - C-X-C Motif Chemokine Ligand 13, YKL-40 - chitanse-3 like-protein-1, BICAMS - Brief International Cognitive Assessment for Multiple Sclerosis, BVMT-R - Brief Visuospatial Memory Test Revised, CVLT - California Verbal Learning Test, SDMT - Symbol Digit Modalities Test, VFT1 - Verbal Fluency Test phonological version, VFT2 - Verbal Fluency Test semantic version, SCWT-A - Stroop Color and Word Test part A, SCWT-B - Stroop Color and Word Test part B.

Group	≥1.0 (EDSS ≤5.5) or ≥0.5 (EDSS >5.5)	OR (95%CI)	p-value
**PMS**	NfL	0.99 (0.97; 1.01)	0.21
	CXCL-13	1.00 (1.00; 1.00)	0.19
	YKL-40	1.00 (1.00; 1.00)	0.21
	BICAMS	0.86 (0.39; 1.87)	0.7
	BVMT-R	0.98 (0.89; 1.08)	0.72
	CVLT	1.02 (0.96; 1.09)	0.49
	SDMT	0.98 (0.92; 1.04)	0.5
	VFT1	1.07 (0.92; 1.23)	0.39
	VFT2	0.96 (0.86; 1.08)	0.51
	SCWT-A	1.03 (0.94; 1.13)	0.56
	SCWT-B	1.02 (0.99; 1.05)	0.19
**SPMS**	NfL	0.98 (0.95; 1.01)	0.26
	CXCL-13	1.00 (0.99; 1.00)	0.21
	YKL-40	1.00 (1.00; 1.00)	0.28
	BICAMS	0.50 (0.14; 1.80)	0.29
	BVMT-R	1.00 (0.84; 1.19)	0.98
	CVLT	1.06 (0.96; 1.17)	0.22
	SDMT	1.08 (0.95; 1.23)	0.21
	VFT1	1.03 (0.85; 1.23)	0.79
	VFT2	1.06 (0.90; 1.25)	0.48
	SCWT-A	1.03 (0.83; 1.26)	0.8
	SCWT-B	1.01 (0.96; 1.06)	0.79
**PPMS**	NfL	0.99 (0.97; 1.02)	0.67
	CXCL-13	1.00 (1.00; 1.00)	0.35
	YKL-40	1.00 (1.00; 1.00)	0.57
	BICAMS	1.25 (0.43; 3.68)	0.68
	BVMT-R	0.98 (0.87; 1.10)	0.69
	CVLT	0.99 (0.90; 1.09)	0.81
	SDMT	0.94 (0.86; 1.02)	0.13
	VFT1	1.14 (0.89; 1.47)	0.29
	VFT2	0.88 (0.73; 1.05)	0.15
	SCWT-A	1.03 (0.92; 1.14)	0.62
	SCWT-B	1.03 (0.99; 1.06)	0.18

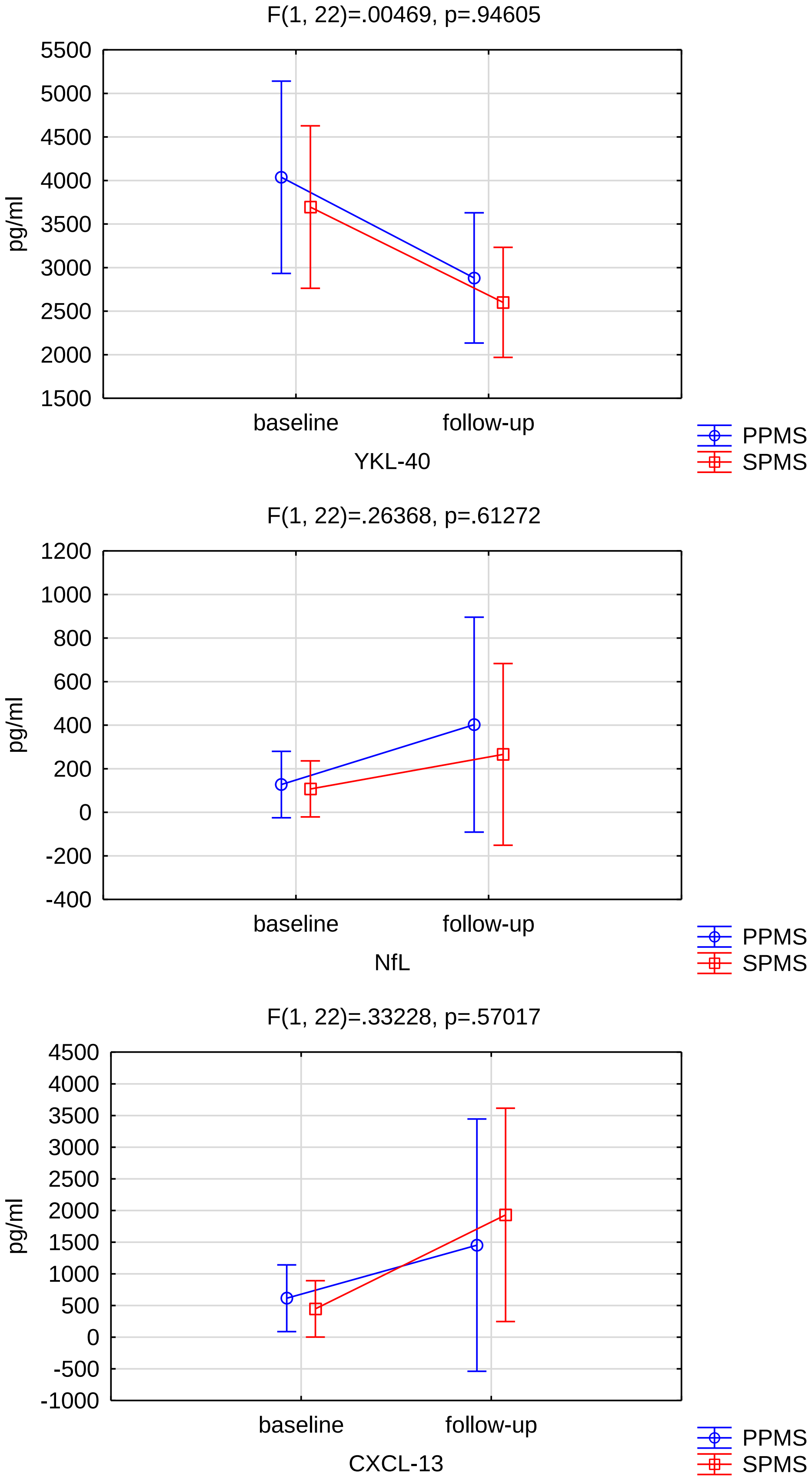

Figure S2. Serum molecular biomarkers at baseline and after follow-up with regard to PMS subtype after exclusion of patients treated with ocrelizumab. Data presented as means with standard error (SE). The values did not reach statistical significance. PPMS - primary progressive multiple sclerosis, SPMS - secondary

progressive multiple sclerosis, YKL-40 - chitanse-3 like-protein-1, NfL - neurofilament light chain, CXCL-13 - C-X-C Motif Chemokine Ligand 13.
